# Telerehabilitation Versus Traditional Care Following Total Hip Replacement: A Randomized Controlled Trial Protocol

**DOI:** 10.2196/resprot.7083

**Published:** 2017-03-02

**Authors:** Mark Nelson, Michael Bourke, Kay Crossley, Trevor Russell

**Affiliations:** ^1^ Physiotherapy Department Queen Elizabeth II Jubilee Hospital Brisbane Australia; ^2^ School of Health and Rehabilitation Sciences University of Queensland Brisbane Australia; ^3^ La Trobe Sport and Exercise Medicine Research Centre College of Science, Health and Engineering La Trobe University Melbourne Australia

**Keywords:** total hip replacement, total hip arthroplasty, telerehabilitation, telehealth, physiotherapy, physical therapy, rehabilitation

## Abstract

**Background:**

Total hip replacement (THR) is the gold standard treatment for severe hip osteoarthritis. Effectiveness of physical rehabilitation for THR patients following discharge from hospital is supported by evidence; however, barriers such as geographical location and transport can limit access to appropriate health care. One solution to this issue is using an alternative model of care using telerehabilitation technology to deliver rehabilitation programs directly into patients’ homes. A telerehabilitation model may also have potential health care cost savings for health care providers.

**Objective:**

This study aims to determine if a telerehabilitation model of care delivered remotely is as effective as face-to-face rehabilitation in the THR population and cost effective for health care providers and patients.

**Methods:**

A total of 70 people undergoing THR will be recruited to participate in a randomized, single-blind, controlled noninferiority clinical trial. The trial will compare a technology-based THR rehabilitation program to in-person care. On discharge from hospital, participants randomized to the in-person group will receive usual care, defined as a paper home exercise program (HEP) targeting strengthening exercises for quadriceps, hip abductors, extensors, and flexors; they will be advised to perform their HEP 3 times per day. At 2, 4, and 6 weeks postoperatively, they will receive a 30-minute in-person physiotherapy session with a focus on gait retraining and reviewing and progressing their HEP. The telerehabilitation protocol will involve a program similar in content to the in-person rehabilitation program, except delivery will be directly into the homes of the participants via telerehabilitation technology on an iPad. Outcomes will be evaluated preoperatively, day of discharge from in-patient physiotherapy, 6 weeks and 6 months postoperatively. The primary outcome will be the quality of life subscale of the hip disability and osteoarthritis outcome score, measured at 6 weeks. Both intention-to-treat and per-protocol analyses as recommended in the extension of the Consolidated Standards for Reporting Trials (CONSORT) guideline for noninferiority trials will be performed.

**Results:**

Recruitment commenced in September 2015 and is expected to be completed by June 2017. Data collection will be completed by December 2017. It is anticipated the results from this trial will be published by July 2018.

**Conclusions:**

Previous research investigating telerehabilitation in postoperative orthopedic conditions has yielded promising results. If shown to be as effective as in-person care, telerehabilitation after THR could be helpful in addressing access issues in this population. Furthermore, it may help reduce the cost of health care provision by enabling patients to take a more independent approach to their rehabilitation.

**Trial Registration:**

Australian New Zealand Clinical Trials Registry ACTRN12615000824561; https://www.anzctr.org.au/Trial/Registration/TrialReview.aspx?id=364010 (Archived by WebCite at http://www.webcitation.org/6oWXweVfI)

## Introduction

Total hip replacement (THR) is the gold standard treatment for severe hip osteoarthritis. A rising trend has seen an increase of approximately 40% in procedures over the last decade, with over 40,000 and 85,000 THRs being performed in Australia and the United Kingdom, respectively, in 2014 [1,2]. This trend is even more pronounced in the United States where the number of THRs performed in patients aged 45 years and over rose from 138,700 in 2000 to 310,800 in 2010 [3]. Optimizing acute postoperative care for THR patients through enhanced and rapid recovery programs has been effective [4-9]. Such programs include preoperative consultation, multimodal analgesia, and early physiotherapy intervention and can decrease length of stay and improve patient outcomes.

Optimal care beyond discharge from hospital remains unknown. Effectiveness of physical rehabilitation for THR patients following discharge from hospital is supported by evidence [10-14]; however, the quality of existing trials prevents robust conclusions on the optimal content of rehabilitation programs. Recommendations regarding content include arm ergometer interval exercise [10], aerobic type dance routines [15], various strength and range of motion (ROM) exercises with and without resistance [11-13,16,17], and walking programs [13,17]. Existing literature also varies with respect to the quantity, timing to commencement, and frequency of physiotherapy rehabilitation [10-13,15-23]. Frequency ranges from twice daily [21] to twice weekly [15], and timing to commencement from immediately following hospital discharge [11] to greater than 6 months after [15,21]. This variability presents difficulties in determining usual physiotherapy care for THR patients after discharge from hospital.

We conducted a national survey of Australian physiotherapists to establish usual physiotherapy care for THR patients following discharge from hospital in Australia. Of 151 physiotherapists representing both public and private facilities and metropolitan and rural areas, 116 (76.8%) responded to the questionnaire. Usual care programs for physiotherapy in Australia consist of 1 to 5 sessions of physiotherapy commencing within 2 weeks of hospital discharge and lasting 4 to 8 weeks. Commonly, physiotherapy sessions included strengthening of hip abductors and extensors and hip and knee flexors; education on hip precautions and exercise progression; gait retraining; stairs practice; and ROM exercises for hip abduction, extension, and flexion. Physiotherapy sessions are complemented by a home exercise program (HEP).

One alternative model of care is the use of telerehabilitation technology to deliver rehabilitation programs directly into patients’ homes. This has the potential benefit of addressing access issues for patients living in rural and remote areas and patients living in urban areas with transport difficulties. Many THR patients find it difficult to access health care once discharged from hospital. The elderly demographic coupled with the risk of THR dislocation postoperatively can make driving and transportation difficult [24]. Access to rehabilitation programs is compounded with the financial cost to the patient [25] and health systems to provide domiciliary services in conjunction with or in lieu of center-based care. For patients living outside urban areas, access issues become magnified due to traveling distances and time for either the patient or treating clinician [26,27]. The use of technology-mediated HEPs may also encourage patients to exercise more frequently, potentially addressing strength deficits documented in the literature in THR patients postoperatively [28]. In addition to addressing access issues, there are potential savings in the cost of health care provision.

Previous research investigating telerehabilitation in postoperative orthopedic conditions has yielded promising results. The majority of research in this area has been focused on the total knee replacement (TKR) population. Multiple randomized controlled trials have compared telerehabilitation programs to conventional programs for postoperative rehabilitation in TKR patients [29-31]. Russell et al [29], Tousignant et al [30], and Piqueras et al [31] all demonstrated the efficacy of telerehabilitation programs compared to in-person programs in the TKR population. In addition to achieving comparable outcomes to conventional rehabilitation, patients participating in telerehabilitation reported high levels of satisfaction with their program [32,33].

This study aims to determine if delivery of usual physiotherapy care via telerehabilitation is as effective as in-person usual physiotherapy care in the THR population and cost effective for health care providers and patients.

## Methods

### Experimental Design

A randomized, single-blinded, controlled noninferiority clinical trial [34] will be conducted comparing a technology-based THR rehabilitation program to in-person care. The trial protocol has been developed conforming to Standard Protocol Items: Recommendations for Interventional Trials (SPIRIT) guidelines.

Ethical approval was obtained from the Metro South Human Research Ethics Committee (HREC No. HREC/14/QPAH/628) and University of Queensland Medical Research Ethics Committee.

### Participants

All patients undergoing primary THR at Queen Elizabeth II (QEII) Jubilee Hospital, Brisbane, Australia, will be approached by a physiotherapist for consent to participate in the study. Consenting patients will have preoperative outcome measures collected. Patients will formally enter the study and be randomized to a group following their operation providing they satisfy the following inclusion and exclusion criteria.

Participants will be eligible for inclusion if they are undergoing primary elective THR, can attend 5 in-person appointments (outcome measure assessment preoperatively, physiotherapy rehabilitation sessions at weeks 2, 4, and 6 postoperatively, and a final outcome measure assessment at 6 months postoperatively), and are able to independently provide signed informed consent.

Participants will be excluded if they have comorbidities preventing participation in rehabilitation (eg, severe obstructive pulmonary disease, hemiplegia following stroke), are undergoing revision THR, experience intraoperative complications that prevent participation in the Queensland Health THR clinical pathway, or are unable to mobilize full weight-bearing in a bipedal manner with or without a walking aid.

**Figure 1:**
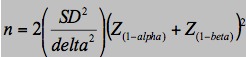
Sample size formula.

Sample size calculations were conducted based on existing data for the subscales of the Hip disability and Osteoarthritis Outcome Score (HOOS) [35]. Required data was available for the pain, symptoms, and quality of life (QOL) subscales. Sample size was calculated using the noninferiority power calculation described by Jones [36] and shown in Figure 1.

Three separate calculations were undertaken for each of the pain, symptoms, and QOL subscales. In the absence of existing delta values from comparable studies, the minimal clinically important improvement (MCII) values determined by Paulsen [37] were applied as our noninferiority delta margin (QOL 17, pain 24, symptoms 23). We argue that if the difference between the groups is less than what has been established as the minimum improvement that matters clinically, we are willing to accept the new intervention as equivalent. Standard deviation values were taken from Duivenvoorden [38], who applied the HOOS to a THR population and reported the standard deviation of the change in HOOS pain, symptoms, and QOL scores from preoperatively to 12 months postoperative (QOL 26.3, pain 22.8, symptoms 14.9). Calculations were based on 80% power and an alpha value of .05. QOL values yielded the largest sample size of 30 per group. Symptoms and pain produced sample sizes of 6 and 12, respectively. Accounting for a 15% dropout rate, a sample size of 35 participants per group equating to a total of 70 participants will be recruited.

### Randomization

Randomization will be performed using a computer random numbers generator to allocate participants to either the in-person (control) group or the telerehabilitation (intervention) group. Randomization will be restricted by a permuted block design of size 4 which will be generated by an independent administrative officer. Randomization codes will be sealed in sequentially numbered opaque envelopes that will be assigned to participants in their order of recruitment by an independent administrative officer.

### Interventions

Interventions will be performed by registered physiotherapists employed by the QEII Jubilee Hospital, Brisbane, Australia. All physiotherapists performing interventions will receive training in the delivery of a standardized exercise program that participants will commence on discharge from hospital. The program will be progressed by the treating physiotherapist based on their assessment and participant progression throughout the 6-week intervention period.

On discharge from hospital, participants randomized to the in-person group will receive usual care, defined as a standardized paper HEP targeting strengthening exercises for quadriceps, hip abductors, extensors, and flexors; they will be advised to perform their HEP 3 times per day and provided with an exercise diary to record exercise compliance. At 2, 4, and 6 weeks postoperatively they will attend QEII Jubilee Hospital physiotherapy outpatient department for a 30-minute in-person physiotherapy session with a focus on gait retraining and reviewing and progressing their HEP.

The telerehabilitation protocol will involve a program similar in content to the in-person rehabilitation program, except delivery will be directly into the homes of the participants via telerehabilitation technology on an Apple iPad. Two apps will be used to deliver this program. Participant’s standardized HEPs will be facilitated using the Wellpepper clinic (Wellpepper Inc, Seattle, WA) app. Wellpepper is an app enabling health care professionals to create exercise programs that patients can follow on a tablet device. The app will provide notifications to complete the exercises and prerecorded videos and instructions of their exercises and enables the patient to record pain levels and difficulty at the conclusion of the exercise. Patients can contact their health care provider through a messaging system built in to the app. The health care professional has a clinic version of Wellpepper installed on an iPad that enables them to review and adjust exercises and communicate with all patients under their care. Real-time video-based physiotherapy consultations will be conducted with the patient via the eHAB app (NeoRehab, Brisbane, Australia). eHAB is a clinically validated telerehabilitation system that allows clinicians to provide services to their patients via real-time videoconferencing into the home.

On discharge from hospital, participants randomized to the telerehabilitation group will be provided an iPad with Wellpepper and eHAB installed. They will undergo training in the use of both apps. All iPads will be enabled with prepaid mobile data.

Participants allocated to the telerehabilitation arm of the trial will receive 3 notifications per day via the Wellpepper app reminding them to perform their exercise program. At the conclusion of each exercise they will be prompted by the app to record pain and difficulty levels experienced during the exercise. Once recorded, this information becomes available to the physiotherapist. The physiotherapist will review the Wellpepper clinic app weekly to review participant’s progress, adjust exercises as required, and respond to any communication via the messaging system. Patients who have not accessed the app and recorded exercises in Wellpepper will be contacted via telephone to discuss their progress. At 2 weeks following hospital discharge, participants will receive a physiotherapy session via real-time videoconferencing using the eHAB app. This session will enable analysis and advice regarding gait retraining and exercise progression. Following this session, participants will continue to use the Wellpepper app to facilitate their rehabilitation until 6 weeks postoperatively. Telerehabilitation participants may receive additional eHAB appointments following their 2-week review if deemed appropriate by the physiotherapist.

Participants from both groups will have outcome measures collected in an in-person assessment at 6 weeks postoperatively. If at this session it is deemed they require further physiotherapy input, they will be booked for an in-person physiotherapy session. The requirement of further physiotherapy will be based on the outcome measures collected by the blinded assessor. The main determinant of additional physiotherapy will be reliance on a walking aid during the timed Up and Go test when participants had mobilized unaided preoperatively. Participants will be instructed not to advise the assessor which group they were randomized to or ask clinical questions of the assessor. Likewise, the assessors will not seek this information from the participant.

### Outcome Assessment

Outcome measures will be collected at 4 time points: preoperatively, day of discharge from in-patient physiotherapy, and 6 weeks and 6 months postoperatively. Following the final physiotherapy session of the 6-week intervention period, 6-week assessments will be conducted. Not all outcome measures will be collected at each time point (Table 1). All assessors will receive training in standardized methods of collecting outcomes. Assessors will conduct blinded assessments of participants from both groups to minimize any assessor effects.

**Table 1:** Summary of outcome measures per time point.

	Time point collected				
Outcome measure	Preoperatively	Discharge from physiotherapy	6 weeks in-person	6 weeks telerehabilitation	6 months
HOOS^a^	x		x	x	x
SF-12^b^	x		x	x	x
EQ-5D-5L^c^	x	x	x	x	x
System usability scale				x	
Patient satisfaction questionnaire			x	x	
Technology preferences	x			x	
Timed Up and Go	x	x	x	x	x
Muscle strength	x		x	x	x
Step test	x		x	x	x
HEP^d^ compliance			x	x	

^a^HOOS: Hip disability and Osteoarthritis Outcome Score.

^b^SF-12: Short Form-12.

^c^EQ-5D-5L: EuroQol 5 dimensions questionnaire.

^d^HEP: home exercise program.

The primary outcome will be the QOL subscale of the HOOS, measured at 6 weeks. The HOOS is a self-administered questionnaire that assesses participants’ opinion about their hip and associated problems and evaluates symptoms and functional limitations related to the hip during a therapeutic process [35]. The HOOS consists of 5 subscales; pain, other symptoms, function in daily living, function in sport and recreation, and hip-related QOL. The last week is taken into consideration when answering the questions. Standardized answer options are given (5 Likert boxes), and each question receives a score from 0 to 4. A normalized score (100 indicating no symptoms and 0 indicating extreme symptoms) is calculated for each subscale. The HOOS has established content validity in a THR population and has high test-retest reproducibility (intraclass correlation coefficient >0.78) [35,39]. Secondary outcome measures include the following series of questionnaires and physical outcomes.

### Questionnaires

The Short Form-12 (SF-12) is a multipurpose, short-form health survey with 12 questions that measures functional health and well-being from the patient’s point of view. It is a generic measure, as opposed to one that targets a specific age, disease, or treatment group [40].

The EuroQol 5 dimension 5 level questionnaire (EQ-5D-5L) is a standardized instrument for use as a measure of health outcome. Participants answer questions regarding mobility, self-care, usual activities, pain/discomfort, and anxiety/depression. Each dimension has 5 levels: no problems, slight problems, moderate problems, severe problems, unable to/extreme problems [41].

Participants will complete a satisfaction questionnaire pertaining to the rehabilitation program they received. This questionnaire is based off the validated health care satisfaction questionnaire developed by Gagnon et al [42].

Participants will complete a self-report questionnaire pertaining to their preferences, access to, and use of technology.

### Physical Outcomes

The timed Up and Go test is a reliable and valid test for quantifying functional mobility and can also be used to follow clinical change over time. The participant rises from an arm chair, walks 3 meters, turns, walks back and sits down again [43].

Hip strength (flexion, extension, abduction, adduction, internal rotation, and external rotation) and knee strength (extension) will be measured (kgs) using a Lafayette 01165 manual muscle tester. All persons performing manual muscle testing will be trained using standardized methods for each muscle group.

The step test involves stepping one foot on, then off, a block as quickly as possible in a set time period. It was originally developed to assess dynamic standing balance in stroke patients [44]. Subsequent studies have proven it a reliable balance outcome measure in both the hip osteoarthritis and postsurgical hip fracture populations [45,46].

### Other Outcomes

The system usability scale is a reliable tool for measuring the usability of a system. Participants are asked to score 10 items with 1 of 5 responses ranging from Strongly Agree to Strongly Disagree. Only participants assigned to the telerehabilitation group will complete the system usability scale [47].

HEP compliance will be collected during the period from hospital discharge to 6 weeks postoperatively. The telerehabilitation group will have this information automatically collected via the Wellpepper app. Participants from the in-person group will be provided with a paper-based exercise diary to complete.

### Blinding

Participants and treating physiotherapists will not be blinded to the allocation. The physiotherapists assessing outcomes at each time point will be blinded. Multiple physiotherapists will be trained in performing outcome assessments to enable compliance with blinding. Physiotherapists who have provided rehabilitation to a participant in either group will not be involved in the outcome assessment of that patient.

### Economic Outcomes

Data will be collected at 4 time points to enable analysis of both patient and health care provider costs: 2 weeks, 4 weeks, 6 weeks, and 6 months postoperatively.

Data relating to patient-associated costs will include time per physiotherapy appointment including travel and associated carer time, cost of travel to and from appointments using distance traveled and per kilometer values provided by the Australian taxation office for private vehicle use or patient-reported public transport and taxi fares if not using a private vehicle, gap fees associated with health care visits related to their hip surgery, and cost of time off work related to their hip surgery using the median Australian wage.

Data relating to health care provider costs will include cost per appointment using standardized government charges for in-person physiotherapy appointments, clinician time multiplied by hourly pay rate for telerehabilitation appointments and time spent monitoring the telerehabilitation system, cost of THR-related health care visits using standardized government charges, and cost of software subscriptions to Wellpepper Inc and eHAB.

### Data Management and Monitoring

Data will be collected directly from participants via paper questionnaires and forms. These will be stored in a locked cabinet within the physiotherapy department at QEII Jubilee Hospital. Data from paper forms will be periodically entered into an electronic spreadsheet in a reidentifiable form. Electronic files will be password protected and stored in secured databases for access by research members only. Information will be kept in a reidentifiable format during data collection. This will enable reviewing of data as required to ensure complete and accurate data sets. On completion of data collection, all information will be converted to a nonidentifiable format.

There will be no external data and safety monitoring board. Data and safety monitoring will be the responsibility of the principal and associate investigators.

### Harms

Participation in this trial will not entail additional risks beyond those associated with standard care. If any adverse events (ie, THR dislocation, deep vein thrombosis) are identified, participants will be advised to attend the emergency department as per usual management of THR patients. Participants will be asked regarding adverse events at each physiotherapy session and outcome assessment. Any adverse events identified by treating physiotherapists or outcome assessors during the 6-month trial period will be reported to the primary investigator. Details of these events will be documented in a logbook. Adverse events will be reviewed on completion of the trial to investigate any trends. Additionally, any participant who is unable to attend a physiotherapy session or outcome assessment appointment will have the reason for being unable to attend recorded.

### Statistical Analysis

All missing data within surveys will be managed as per developer guidelines. Both intention-to-treat and as-treated analyses as recommended in the extension of the Consolidated Standards of Reporting Trials (CONSORT) guideline for noninferiority trials will be performed [48]. Prior to statistical analysis, data will be tested for compliance with the assumptions of parametric statistics (normality, skewing, kurtosis, etc). If failing to meet these assumptions, data transformation will be attempted to achieve compliance. Nonparametric equivalents will be employed if parametric assumptions are not met. Covariance will be determined and incorporated into analyses as appropriate. The treatment effect of each intervention will first be computed by comparing the pre-to-post intervention measures. Our primary analysis of noninferiority will be implied if the lower limit of a 1-sided 95% confidence interval of the difference between the telerehabilitation and control group is within the prestated MCII values [37]. Secondary analysis with a linear mixed model (LMM) will be used to ensure no statistically significant differences exist between groups. LMM is appropriate for comparing means in independent samples and has the added advantage of adjusting for baseline differences and being tolerant of missing data. Because baseline differences are adjusted for with this approach, it is possible to compare pre- and posttreatment scores between groups, rather than their change scores. The statistic will be computed with observed outcomes as the dependent variables and with fixed factors of treatment group (telerehabilitation, in-person) and assessment time (preoperatively, day of discharge, 2 weeks, 6 weeks, and 6 months postoperatively). Interactions among these factors will also be assessed. Fixed predicted values and residuals from these analyses will be used for data inspection purposes. The outcome of primary interest is the interaction effect between group and time. An alpha level of .05 will be used for the analysis.

### Economic Evaluation

Concurrently undertaking an economic evaluation with a randomized controlled trial allows for efficient and simultaneous collection of relevant clinical and economic outcomes to be included in both analyses [49]. Given that a noninferiority research hypothesis is proposed, the planned economic evaluation will be a cost-minimization analysis. This analysis answers the question of which health program uses the lower quantity of resources to achieve the same health outcome. Direct costs to the health system and total direct costs (including non–health care costs and out of pocket costs) will be considered in this evaluation. However, an incremental cost-utility analysis (CUA) will be undertaken if a difference in clinical outcomes between groups is determined. The EQ-5D scores will be used to generate quality-adjusted life year scores for the purpose of the CUA. Multivariate sensitivity analyses will be conducted to confirm stability of results and adjust for uncertainty in clinical and economic data [49]. The time horizon for this economic evaluation will be the 6-month follow-up during which each participant is involved in the study, and the base year will be 2015.

## Results

This trial received a Aus $10,000 grant from the Queensland Orthopaedic Physiotherapy Network to fund the infrastructure required to conduct the trial. The trial is being conducted by means of an in-kind contribution from the QEII Jubilee Hospital Physiotherapy Department. Recruitment commenced in September 2015 and is expected to be completed by June 2017. Data collection will be completed by December 2017. It is anticipated the results from this trial will be published by July 2018.

## Discussion

THR is a high-volume surgery with good success rates. Evidence suggests physical rehabilitation is an important component of recovery; however, access to rehabilitation is often limited. This study will investigate the effectiveness of a telerehabilitation program for THR patients once discharged from hospital. It will compare self-reported and physical outcomes from the intervention group to a control traditional-care group. The intervention group will undertake their rehabilitation at home using 2 existing rehabilitation apps (Wellpepper and eHAB) via an iPad. The control group will receive traditional in-person rehabilitation.

The positive results from TKR telerehabilitation studies suggest that similar results could be achieved in the THR population where rehabilitation programs have a focus on functional activities, exercise, and education. If shown to be as effective as in-person care, telerehabilitation for THR patients could help solve an access issue that exists for many of the population. Furthermore, it may help reduce the cost of health care provision by enabling patients to take a more independent approach to their rehabilitation.

## Abbreviations

CONSORT: Consolidated Standards of Reporting Trials
CUA: cost-utility analysis
HEP: home exercise program
HOOS: Hip disability and Osteoarthritis Outcome Score
LMM: linear mixed model
MCII: minimal clinically important improvement
QEII: Queen Elizabeth II
QOL: quality of life
ROM: range of motion
SPIRIT: Standard Protocol Items: Recommendations for Interventional Trials
THR: total hip replacement
TKR: total knee replacement
